# Association between roxadustat use and suppression of thyroid function: a systematic review and meta-analysis

**DOI:** 10.1186/s40780-024-00351-z

**Published:** 2024-06-08

**Authors:** Yuki Nakano, Satoru Mitsuboshi, Kazuhiro Tada, Kosuke Masutani

**Affiliations:** 1 Department of Pharmacy, Saiseikai Futsukaichi Hospital, Fukuoka, Japan; 2 Department of Pharmacy, Kaetsu Hospital, Niigata, Japan; 3 Division of Nephrology and Rheumatology, Department of Internal Medicine, Faculty of Medicine, Fukuoka University, Fukuoka, Japan

**Keywords:** Adverse drug event, Thyroid function, Hypothyroidism, Meta-analysis, Roxadustat, Risk assessment

## Abstract

**Background:**

Based on several case reports and observational studies, there is a growing concern regarding the potential association between roxadustat, a hypoxia-inducible factor prolyl-hydroxylase inhibitor, and suppression of thyroid function. In this systematic review and meta-analysis (PROSPERO: CRD42023471516), we aimed to evaluate the relationship between roxadustat use and suppression of thyroid function.

**Methods:**

We conducted a comprehensive search of MEDLINE via PubMed, ClinicalTrials.gov, and the Cochrane Central Register of Controlled Trials databases using the search term “roxadustat” to identify all relevant studies. The study population comprised adults with renal anemia who participated in a randomized controlled trial or observational study, with roxadustat as the intervention and a placebo or erythropoiesis-stimulating agent (ESA) as the comparator. The primary outcome was suppression of thyroid function and the secondary outcome was hypothyroidism. A meta-analysis was conducted using the DerSimonian–Laird random effects model based on the size of the intention-to-treat population, and the odds ratio (OR) and 95% confidence interval (CI) were calculated. Two reviewers independently screened the articles, extracted data, and assessed studies using the ROBINS-I tool.

**Results:**

Of the six studies eligible for inclusion, a meta-analysis was performed using data from two observational studies comparing roxadustat and ESA. The meta-analysis showed that the incidence of suppression of thyroid function was significantly higher with roxadustat use than with ESA use (OR: 6.45; 95% CI: 3.39–12.27; *I*^2^ = 12%). Compared with ESA, roxadustat seemed to potentially increase the risk for suppression of thyroid function in patients with renal anemia.

**Conclusions:**

Our findings highlighted the importance of monitoring thyroid function in patients treated with roxadustat. The results of this review may enhance the safety of using roxadustat to treat renal anemia through advance recognition of the risk for suppression of thyroid function.

**Supplementary Information:**

The online version contains supplementary material available at 10.1186/s40780-024-00351-z.

## Background

Globally, chronic kidney disease (CKD) affects over 800 million people, representing an estimated prevalence of ≥ 10% of the population [1]. Renal anemia is a common secondary complication of CKD that affects over half (53%) of all patients with stage 5 CKD and correlates with an increased risk of cardiovascular events and mortality [2]. Treating renal anemia to reduce the risk of cardiovascular events is recommended by the European Renal Association [3] and constitutes a crucial aspect of managing patients with CKD [4].

Renal anemia is caused by erythropoietin deficiency or functional iron deficiency and is treated using iron agents, erythropoiesis-stimulating agents (ESAs), or hypoxia-inducible factor prolyl-hydroxylase (HIF-PH) inhibitors [5, 6]. HIF-PH inhibitors are a novel class of medication that improves renal anemia by increasing endogenous erythropoietin production or the number of erythropoietin receptors and modulates iron metabolism [7, 8]. However, the use of HIF-PH inhibitors is associated with adverse effects, such as malignancy, retinopathy, and infection [9]; therefore, patient monitoring is important to enable early detection of adverse effects.

Hypo-responsive renal anemia to ESA poses a higher mortality risk if not properly managed and may be effectively treated with HIF-PH inhibitors [5, 10]. Among HIF-PH inhibitors, roxadustat has a long half-life in blood and can be administered orally three times a week, making it particularly useful in the management of renal anemia [11]. However, some cases of roxadustat-associated suppression of thyroid function have been reported [12, 13]. A study using the Japanese Adverse Drug Event Report database reported that only roxadustat, one of the five HIF-PH inhibitors approved in Japan, showed signals associated with suppression of thyroid function [14, 15]. In fact, although hypothyroidism induced by roxadustat was added to the serious adverse reactions section of the Japanese package insert in November 2022, it is notable that suppression of thyroid function by roxadustat has been reported to be frequently asymptomatic [16]. Suppression of thyroid function, even if asymptomatic, is a significant adverse event in renal anemia because thyroid hormones act to increase HIF-1α [17]. Therefore, evaluating existing studies on roxadustat use and thyroid function will facilitate its appropriate usage in managing renal anemia. This systematic review and meta-analysis aimed to evaluate the association between roxadustat use and suppression of thyroid function.

## Methods

### Study design

We conducted a systematic review and meta-analysis of randomized controlled trials (RCTs) and observational studies to evaluate whether roxadustat increases the risk of suppression of thyroid function in patients with CKD. This study was conducted in accordance with the Preferred Reporting Items for Systematic Reviews and Meta-Analyses (PRISMA) guidelines, and the Cochrane Collaboration Handbook (Supplementary Table 1) [18]. The study protocol was registered with PROSPERO (CRD42023471516).

### Search strategy and selection of studies

The study population consisted of adults with renal anemia. The study types included all RCTs, irrespective of whether they had a cluster- or crossover-randomized design, and observational studies. The intervention examined was roxadustat, with the comparator being placebo or ESA.

The following types of studies were excluded: case reports, case series, review articles, studies that did not focus on renal anemia, studies of patients with serious underlying conditions that required organ transplantation, and studies with no assessment of adverse events. There were no restrictions on medication dosage, follow-up period, publication date, or language of publication. If the published report did not cover all outcomes, we contacted the authors to request additional unpublished data.

We conducted a comprehensive search of MEDLINE via PubMed, ClinicalTrials.gov, and the Cochrane Central Register of Controlled Trials (CENTRAL) databases to identify all relevant studies. The search strategy included “roxadustat” [All Fields] in MEDLINE via PubMed, “roxadustat” [All Text] in CENTRAL, and (intervention/treatment: roxadustat) in the ClinicalTrials.gov database. All searches were conducted on October 22, 2023. We conducted full-text reviews of articles with titles and abstracts identified as appropriate based on the inclusion and exclusion criteria. Subsequently, we performed outcome measurement, data extraction, data synthesis, statistical analysis, and quality assessment. The reviews were performed independently by two researchers. Any discrepancies in the evaluation results were discussed to reach consensus.

### Data extraction and quality assessment

The following data were extracted for each study: publication status, intervention details, inclusion and exclusion criteria, conflicts of interest, study period, number of outcome events, number of participants lost to follow-up, authors, country, study design, dosage of roxadustat or ESA, number of participants, population, and average dosing period. The primary outcome was suppression of thyroid function and the secondary outcome was hypothyroidism. This outcome event was retrieved from the results section of the article, or the ClinicalTrials.gov description. Suppression of thyroid function was included based on statements such as “decreased thyroid-stimulating hormone [TSH]”, “FT4 decreased”, or “FT3 decreased”.

The risk of bias for all outcomes in each study was assessed using the Risk Of Bias In Non-randomized Studies of Interventions tool (ROBINS-I) for observational studies [19]. Publication bias was assessed using a funnel plot.

### Data synthesis and statistical analysis

Meta-analysis was performed using the DerSimonian–Laird random effects model based on the size of the intention-to-treat population, and the odds ratio (OR) and 95% confidence interval (CI) were calculated [20]. The heterogeneity of the selected studies was assessed using the Cochrane χ^2^ test, τ^2^, and *I*^2^ statistics.

Statistical significance was set at p-value < 0.05, unless otherwise specified. Data analysis was performed using the “meta” package of R software (version 4.2.2 R Core Team 2022; Vienna, Austria; https://www.R-project.org/).

## Results

### Description of selected studies

**Fig. 1:**
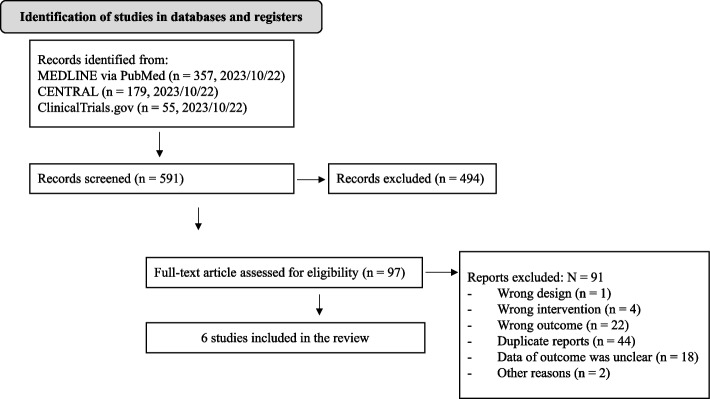
Preferred reporting items for systematic reviews and meta-analysis flow diagram. CENTRAL, Cochrane Central Register of Controlled Trials; MEDLINE, Medical Literature Analysis and Retrieval System Online

We identified 591 studies from electronic databases: 357 in PubMed, 179 in CENTRAL, and 55 in ClinicalTrials.gov. Screening of the titles and abstracts initially identified 97 studies. Subsequently, a full-text review led to the exclusion of 91 studies with 44 duplicate reports, 22 with wrong outcomes, 18 with unclear outcome data, four with wrong interventions, one for wrong design, and two for other reasons (retraction of the article and incomplete clinical trial). Finally, six studies were included in the review (Fig. 1). Table 1 presents the characteristics of the included studies, which were published between 2015 and 2023. Data on hypothyroidism were extracted from the results sections and supplementary materials of the included RCTs [21–24]. For observational studies, suppression of thyroid function was identified based on a decrease in TSH, FT3, or FT4 [25], or a ≥ 50% decrease in TSH [26]. The number of cases was too small to perform meta-analysis in RCTs with placebo or ESA as comparators [21, 22]. Therefore, the meta-analysis was performed on two observational studies, with ESA as the comparator [25, 26], and supplemented by two RCTs with the same comparator for complementary analysis [23, 24].

**Table 1:** Summary of included studies

Author/Year	Country	Study design	Roxadustat dose	Comparator dose	The number of patients (hypothyroidism or suppression of thyroid function) with roxadustat vs. comparator	Population	Follow-up period
Comparator: Placebo							
Besarab et al. (2015) [21]	United States	RCT (Single-blind)	0.7–2.0 mg/kg/day	Placebo	88 (1) vs. 28 (0)	NDD-CKD	4 weeks
Coyne et al. (2020) [22]	United States	RCT (Double-blind)	70 mg/day (45–70 kg) or 100 mg/day (≥ 70 kg)^a^ TIW	Placebo	616 (0) vs. 306 (1)	NDD-CKD	1.4 years
Comparator: ESA							
Charytan et al. (2021) [23]	United States	RCT (Open-label)	70–200 mg/day TIW^a^	Epoetin alfa 6000–16000 IU/week^a^	370 (1) vs. 370 (0)	DD-CKD	1–3 years
Fishbane et al. (2022) [24]	United States	RCT (Open-label)	70 mg (45–70 kg) or 100 mg (> 70–160 kg) TIW^a^	Epoetin alfa 50 IU/kg TIW^a^	1048 (1) vs. 1053 (0)	DD-CKD	1.8 years
Zheng et al. (2023) [25]	China	Observational study	Standard doses^b^	rHuEPO standard doses^c^	50 (36) vs 60 (21)	NDD- or DD-CKD	1 year
Cheng et al. (2023) [26]	China	Observational study	Standard doses^b^	rHuEPO standard doses^c^	76 (37) vs 75 (7)	NDD- or DD-CKD	0.25–1.25 year

Hypothyroidism in RCTs was identified from the results sections and supplementary materials of the included studies [21–24]. For observational studies, suppression of thyroid function was identified based on a decrease in TSH, FT3, or FT4 [25], or a ≥ 50% decrease in TSH [26]

DD-CKD, dialysis-dependent (hemodialysis or peritoneal dialysis) chronic kidney disease; ESA, erythropoiesis-stimulating agent; NDD-CKD, non-dialysis-dependent chronic kidney disease; RCT, randomized controlled trial; rHuEPO, recombinant human erythropoietin; TIW, thrice weekly

^a^It was equal to or equivalent to the pretrial dose and the dose was subsequently adjusted according to an algorithm

^b^Standard doses in China are 100 mg (45–60 kg) or 120 mg (≥ 60 kg) for DD-CKD and 70 mg (45–60 kg) or 100 mg (≥ 60 kg) for NDD-CKD, three times a week. Moreover, the maximum dose is 2.5 mg/kg/dose

^c^The standard dose of rHuEPO in China is 100–150 IU/kg/week in DD-CKD and 75–100 IU/kg/week in NDD-CKD. The dose can be increased to 30 IU/kg/week for epoetin alfa and 60–240 IU/kg/week for epoetin beta

### Quality assessment

The risk of bias was assessed for the two observational studies that were included in the meta-analysis. The overall risk of bias of the two observational studies was assessed as “Serious”. This was because domains 1 to 3 were judged “Serious” owing to unadjusted confounding and the presence of patient selection and intervention bias (Table 2). Although the funnel plot suggested publication bias (Supplementary Fig. 1), it was difficult to interpret owing to the limited number of eligible studies [25, 26]. The risk of bias for the two RCTs in the complementary analysis also was assessed as “High” (Supplementary Table 2) [23, 24].

**Table 2:** Risk of bias summary for the observational studies

Author, year	Bias domain: Cochrane risk-of-bias assessment tool for non-randomized trials							Overall
	**Domain 1**	**Domain 2**	**Domain 3**	**Domain 4**	**Domain 5**	**Domain 6**	**Domain 7**	
Zheng et al. (2023) [25]	Serious	Serious	Serious	Moderate	Moderate	Moderate	Moderate	Serious
Cheng et al. (2023) [26]	Serious	Serious	Serious	Moderate	Moderate	Moderate	Moderate	Serious

Domain 1: bias owing to confounding, Domain 2: bias owing to selection of participants, Domain 3: bias in classification of interventions, Domain 4: bias owing to deviations from intended interventions, Domain 5: bias owing to missing data, Domain 6: bias in measurement of outcomes, Domain 7: bias in selecting the reported result

### Meta-analysis

**Fig. 2:**
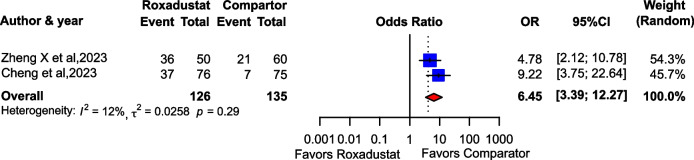
Forest plot comparing the suppression of thyroid function incidence in patients treated with roxadustat and an erythropoiesis-stimulating agent. CI, confidence interval; ESA, erythropoiesis-stimulating agent; OR, odds ratio

Two observational studies with a total of 261 participants (in which the comparator was ESA) were included in the meta-analysis. Suppression of thyroid function was observed in 73 of 126 patients (57.9%) treated with roxadustat and 28 of 135 patients (20.7%) treated with ESA. The risk of hypothyroidism was significantly higher in patients treated with roxadustat than in those treated with ESA (OR: 6.45; 95% CI: 3.39–12.27; *I*^2^ = 12%; Fig. 2). In addition, a meta-analysis including two RCTs were also performed with similar results (OR: 6.11; 95% CI: 3.41–10.94; *I*^2^ = 0%; Supplementary Fig. 2).

## Discussion

To the best of our knowledge, this is the first systematic review and meta-analysis suggesting that roxadustat may increase the risk for suppression of thyroid function compared with ESA. Roxadustat may act as a selective agonist of the thyroid hormone receptor due to its structural similarity to triiodothyronine [27]. This can lead to a decrease in triiodothyronine or thyroxine levels through negative feedback on pituitary TSH-producing cells, even without excess thyroid hormone levels [28]. Two case reports have shown that both suppression of thyroid function and hypothyroidism can be attributed to roxadustat [12, 13]. Furthermore, the cases that developed to hypothyroidism had underlying with thyroiditis. Recently, comparisons with daprodustat suggest that suppression of thyroid function is a specific adverse event associated with roxadustat. These events did not appear to affect the lipid profile, indicating that they are less likely to develop into hypothyroidism [16]. Although it remains unclear which patients may develop hypothyroidism with roxadustat use, caution might be warranted for those with thyroid-related diseases or in cases of dose titration or high dose as roxadustat shows dose-dependent inhibition of thyroid function in vitro and previous case report [13, 27].

The meta-analysis of observational studies included in this study showed a risk of suppressed thyroid function; however, the actual number of reported cases of hypothyroidism was so small that a meta-analysis could not be performed. In the four RCTs, three cases of hypothyroidism were reported in the roxadustat-treated group and one case was reported in the comparator treatment group, consistent with the overall results. The observational studies included in the analysis of this study reported an incidence of hypothyroidism of 9.3% [26] and 35.0% [25] in the ESA group, aligning with findings of prior studies [29]. Considering these results, it is reasonable to analyze the suppression of thyroid function by roxadustat in a meta-analysis of observational studies. Meanwhile, as RCTs included the collection of adverse events, routine monitoring for roxadustat-induced hypothyroidism is unlikely unless the risk is recognized. Thus, RCTs are likely to collect data on symptomatic rather than asymptomatic hypothyroidism in CKD patients. The results support the hypothesis that roxadustat may suppress thyroid function, although it is less likely to cause hypothyroidism [16].

Two important points regarding the risk of bias should be considered when interpreting the results of this study. First, the observational study included in this review may have been subject to bias owing to confounding factors, participant selection, and the classification of the interventions. It was not possible to completely match the backgrounds of the patients in the roxadustat and ESA groups owing to the nature of the observational study; therefore, there is a risk of residual confounding. Second, participant selection and the classification of interventions included the possibility of treating patients with ESA resistance with roxadustat. ESA resistance is reportedly associated with an increased risk of hypothyroidism; therefore, there was a risk of selection bias [30].

This systematic review and meta-analysis had several limitations. First, placebo- and ESA-controlled trials for the RCTs could not be performed in the meta-analysis because only one case of hypothyroidism was reported in each group, but this low incidence may reflect the low chance of development of hypothyroidism with roxadustat. Second, the two observational studies included in the meta-analysis of this study had various risks of bias. Nevertheless, our findings should be sufficient to highlight the importance of monitoring for suppression of thyroid function in roxadustat use, even in the presence of these biases. Third, the small number of studies included in the meta-analysis precluded a sensitivity analysis. Finally, owing to the small number of studies included in the meta-analysis, it was not possible to evaluate whether there was a dose–response relationship between roxadustat and suppression of thyroid function, or whether the risk of suppression of thyroid function was related to the severity of renal anemia, and future studies should be conducted to elucidate these potential relationships.

## Conclusions

Our findings highlighted the importance of monitoring thyroid function in patients treated with roxadustat. Therefore, physicians treating patients with roxadustat may need to consider regular monitoring of thyroid function. The results of this review may contribute to enhancing the safety of roxadustat use in treating renal anemia by providing advance recognition of the associated risk of hypothyroidism. However, further studies are needed to confirm the association between roxadustat use and hypothyroidism in patients with renal anemia given the limitations of the studies included in the meta-analysis and the limited number of studies available.

### Supplementary Information

Additional file 1: Supplementary table 1. Checklist of items to include when reporting a systematic review (with or without meta-analysis).

## Data Availability

The datasets used and analyzed during the current study are available from the corresponding author on reasonable request.

## Abbreviations

CENTRAL: Cochrane Central Register of Controlled Trials
CI: Confidence interval
CKD: Chronic kidney disease
ESA: Erythropoiesis-stimulating agent
HIF-PF: Hypoxia-inducible factor prolyl-hydroxylase
OR: Odds ratio
PRISMA: Preferred Reporting Items for Systematic Reviews and Meta-Analyses
RCT: Randomized controlled trial
ROBINS-I: Risk Of Bias In Non-randomized Studies of Interventions tool
TSH: Thyroid-stimulating hormone
